# Mind, machine, and the law: reimagining neurotechnology governance through disability rights

**DOI:** 10.1093/jlb/lsag011

**Published:** 2026-05-14

**Authors:** Francisco Bariffi

**Affiliations:** Institute of Human Rights ‘Gregorio Peces-Barba’, University Carlos III of Madrid, Spain

**Keywords:** neurotechnologies, disability rights, cognitive liberty, legal capacity, mental privacy, CRPD (Convention on the Rights of Persons with Disabilities)

## Abstract

The convergence of neurotechnologies and disability raises urgent questions about autonomy, mental integrity, and legal capacity for persons with disabilities. This article examines the human rights implications of emerging neurotechnologies—from brain–computer interfaces to cognitive monitoring tools—through the lens of the United Nations Convention on the Rights of Persons with Disabilities (CRPD). Drawing on historical abuses under the medical model of disability, it argues that the uncritical deployment of neurotechnologies risks replicating patterns of coercion, paternalism, and exclusion. By advancing a normative framework rooted in the CRPD and the social model of disability, the article proposes legal and ethical safeguards to protect mental privacy, ensure informed consent, and affirm supported decision-making. It calls for regulatory and design paradigms that shift from enhancement and correction to inclusion and empowerment. Ultimately, the article contends that disability rights must be at the center of neurotechnologies governance to prevent ableist harms and foster equitable innovation.

## I. INTRODUCTION

We are entering a new technological era in which the capacity to access, decode, and even influence human thought has shifted from the realm of speculative fiction to the concrete domain of scientific possibility. Neurotechnologies—ranging from brain–computer interfaces to artificial intelligence (AI)-powered brain and behavioral monitoring systems—such as electroencephalography (EEG)-based neuroimaging and affective computing tools that infer cognitive or emotional states—are rapidly transforming not only medical and scientific practices but also our collective understanding of cognition, identity, and autonomy. These developments compel a fundamental reassessment of how law, ethics, and human rights should respond to the evolving frontier of brain-based innovation.

For the purposes of this article, ‘*neurotechnologies*’ refer to tools and systems that directly record, decode, or modulate neural activity in order to assess, enhance, or alter cognitive and affective processes. These include both invasive devices—such as deep brain stimulation (DBS) implants and intracranial brain–computer interfaces (BCIs)—and non-invasive technologies, such as EEG, transcranial magnetic stimulation, and functional magnetic resonance imaging (fMRI). The term also extends to hybrid AI-based systems that infer mental states through algorithmic analysis of neural or behavioral data.

This article addresses these transformations through the prism of disability rights and the United Nations Convention on the Rights of Persons with Disabilities (CRPD). Far from being peripheral subjects in the neurotechnological debate, persons with disabilities—particularly those with intellectual or psychosocial disabilities—occupy a central position in discussions about agency, consent, integrity, and inclusion. Historically subjected to paternalistic interventions and medical experimentation under the guise of care, persons with disabilities now face a renewed, technologically enhanced set of challenges that test the limits of legal capacity, bodily autonomy, and mental privacy.

Yet the stakes go beyond the risk of misuse. At the core lies a normative question: who defines what counts as normal cognition, acceptable behavior, or legitimate intervention? Neurotechnologies, with their capacity to modulate neural function and predict mental states, do not merely carry medical potential—they also encode social values, medical paradigms, and normative judgements about what minds and bodies should be. Without rigorous ethical and legal frameworks, these tools risk reinforcing ableist assumptions, reviving eugenic logics, and exacerbating existing inequalities in access and participation.

Anchored in the social model of disability and the transformative framework of the CRPD, this article examines how neurotechnologies intersect with core human rights principles, including dignity, equality, autonomy, integrity, privacy, and liberty. It argues that the future of neurotechnologies must be guided not by an imperative to ‘fix’ disability but by a commitment to dismantling structural barriers and enabling diverse modes of existence. The CRPD offers more than legal protection—it reorients our ethical compass, urging us to construct a technological future where human difference is neither pathologized nor erased but respected as a foundation of justice, understood as the fair recognition and accommodation of human diversity. By interrogating the promises and perils of neurotechnologies through the lens of the CRPD, this article contributes to a growing body of critical legal scholarship that insists on the full participation of persons with disabilities in shaping the policies, ethics, and technologies that affect their lives. It is a call to resist new forms of neurological paternalism and to embed in our neurotechnological futures the very principles that animate the disability rights movement: autonomy, inclusion, and the inalienable worth of every human mind.

To appreciate the stakes of this transformation, it is crucial to examine how neurotechnologies already intersect with the lived experience of disability and the practical challenges of inclusion.

## II. NEUROTECHNOLOGIES, HISTORICAL MISTRUST, AND THE AMBIVALENCE OF TECHNOLOGICAL PROGRESS

The relationship between neurotechnological innovation and disability cannot be understood without considering the historical context in which scientific and medical interventions have shaped perceptions of impairment. From early institutional practices to modern brain-based devices, a persistent tension endures between technological promise and the memory of coercion.

Neurotechnologies are increasingly influencing the ways in which we conceive disability and the options available to address it.^1^ By restoring sensory capabilities, enriching communication, and alleviating movement or cognitive impairments, these devices hold great promise for persons with disabilities.^2^ Cochlear implants, for instance, can promote social engagement for those with profound hearing impairments;^3^ retinal implants can offer partial vision to individuals with certain types of blindness;^4^ BCIs provide a means for people with severe motor impairments to operate robotic limbs or digital interfaces;^5^ and DBS, initially created to reduce Parkinsonian tremors, has been extended to treat serious depression and obsessive-compulsive disorder.^6^

It is nonetheless impossible to view these breakthroughs in isolation from the broader history of disability-related technologies.^7^ Early interventions for persons with disabilities often followed a medical model that emphasized ‘correcting’ impairments via prosthetics, orthopedic solutions, and rehabilitation methods, framing the disabled body as an object of intervention rather than a source of intrinsic worth and self-determination.^8^ Yet, as growing calls for social and structural reform gathered strength in the late twentieth century, assistive technologies began to align with the social model of disability, focusing on improved communication, mobility, and autonomy, rather than rigidly aiming to remediate impairments.^9^

However, the historical trajectory of medical and technological ‘solutions’ reveals how the pursuit of normalization has often carried the seeds of control. During much of the nineteenth and twentieth centuries, persons with disabilities were subjected to a distinctly paternalistic approach known as the medical model.^10^ This perspective framed disability as a pathological anomaly, calling for corrective or eliminative interventions that prioritized professional expertise over personal autonomy.^11^

Early asylum practices captured these ideas. Though ostensibly conceived for protection and rehabilitation, asylums frequently devolved into sites of indefinite confinement, where coercive measures—including forced institutionalization, electroconvulsive therapy, and even lobotomies—were justified under the label of care.^12^ Promoted as therapeutic milestones, these interventions often undermined persons with disabilities’ autonomy and, in numerous instances, led to long-term harm.^13^

Philosopher Michel Foucault critically examined such institutional settings, arguing that ostensibly benign medical structures could become formidable instruments of social control.^14^ By casting persons with disabilities as subjects of professional intervention, these institutions regularly divested individuals of agency. Portraying disability as a deviation demanding eradication effectively concealed the broader social inequalities that obstructed the inclusion of those labeled ‘abnormal’.^15^ The eugenics movement epitomized the extremes of these views, featuring forced sterilizations upheld by theories of genetic ‘improvement’.^16^ Such strategies predominantly targeted individuals with cognitive or psychosocial disabilities, along with marginalized minority groups, fueling a deep-rooted skepticism about medical institutions that has persisted into the twenty-first century.^17^

The memory of such abuses continues to inform contemporary attitudes toward neurotechnologies.^18^ Although lobotomies and involuntary sterilizations may seem relics of a distant past, their ethical reverberations resonate in modern contexts where new interventions promise to ‘repair’ or ‘enhance’ the mind.^19^ Many disability advocates therefore approach neurotechnological innovation with warranted caution, recalling earlier epochs when scientific enthusiasm eclipsed individual rights.^20^

Despite this shift, technology retains a dual character. Past attempts to ‘normalize’ disabled bodies or minds have, on occasion, veered into coercion or entrenched stereotypes, exemplified by forced medical regimes and eugenic projects.^21^ These warnings highlight the complex interplay between social values and innovation. Ethical and social questions also come to the fore because neurotechnologies often entail data collection and potential manipulation of neural signals. The closer a device gets to the brain, the greater the implications for privacy and mental autonomy,^22^ a concern that may be especially pressing for those with intellectual or psychosocial disabilities, given frequent obstacles to informed consent. Additionally, affordability limitations and infrastructure requirements may hinder the availability of advanced neurotechnologies, thus compounding existing disparities. There is also a risk of fostering ableist ideals when the chief objective is to ‘fix’ a person’s impairment at the expense of incorporating user insights or acknowledging the structural barriers that inhibit full participation.^23^

This ambivalence—between emancipation and control—remains central to disability politics in the neurotechnological age. The same devices that can extend communication or mobility can also reproduce dependence if framed within paternalistic models. The historical memory of medical dominance explains why skepticism toward neurotechnological ‘solutions’ is not resistance to progress but a demand for inclusion, consent, and rights-based governance.

The mistrust deriving from these past abuses constitutes a significant factor for both current and future neurotechnological applications. Despite their potentially transformative benefits, neurotechnological innovations risk being viewed with caution if portrayed chiefly as ‘corrective’ or applied within a framework of paternalistic presuppositions.^24^ Caution toward neurotechnological innovation should therefore not be misconstrued as technophobia. Given this legacy, critical vigilance is an ethical necessity, ensuring that enthusiasm for innovation does not eclipse scrutiny of power and consent. What endangers progress is not prudent skepticism but the absence of participatory oversight.

In addition, the fear that newly emerging methods—including AI-driven brain monitoring systems—might facilitate more subtle forms of surveillance or coercion sustains long-held anxieties around autonomy and dignity.^25^ The recurrent specter of misuse echoes the clear legacy of historical interventions that were grounded in the belief that disability reflected an individual failing rather than a socially constructed challenge.

This historical evolution from coercive treatment to rights-based advocacy situates current neurotechnological debates within a broader human-rights frame.

## III. THE SOCIAL MODEL AND THE CRPD

By the late twentieth century, an expanding global movement of disability activists, scholars, and allies began to question the notion that disability resided solely within the individual.^26^ Rather than treating physical, sensory, or cognitive impairments as pathologies to be ‘fixed’, this movement contended that the most disabling elements were social, environmental, and attitudinal barriers. This conceptual overhaul—called the ‘social model of disability’—marked a decisive shift away from the deficit-based lens of the medical model. In this social model, the environment, rather than the body, becomes the key site for reform, prompting efforts to tackle inaccessibility, stigma, and systemic inequalities, instead of aiming simply to correct individual impairments.^27^

This reconfiguration took its impetus from grassroots activism in countries such as the UK and the USA, where persons with disabilities and their allies organized against institutional confinement and inaccessible public facilities.^28^ Their claim, though straightforward, had radical implications: disability results from societal choices—designing buildings without ramps, for example, or using communication strategies ill-suited to those with sensory impairments—rather than from an inherent flaw within the individual. Activists challenged paternalistic approaches, arguing that technological or medical solutions alone would always remain insufficient unless society also addressed exclusionary practices, prejudice, and systemic neglect.^29^

This increasing recognition of the primacy of social barriers set the stage for the unprecedented consolidation of disability rights in a legally binding international instrument.^30^ The United Nations CRPD, adopted in 2006,^31^ epitomizes decades of disability advocacy and scholarship. By enshrining the social model in international law, the CRPD obliges states to combat discrimination holistically, requiring that they remove physical and attitudinal barriers throughout all spheres of life, including employment, education, healthcare, and political engagement.^32^ It promotes the principle of inclusive equality—a concept demanding proactive measures to address barriers so that all people, regardless of impairment, can engage meaningfully in society on an equal basis with others.^33^

Importantly, the CRPD reaffirms that persons with disabilities hold the same rights to autonomy and legal capacity as anyone else, stressing the need to move beyond substituted decision-making systems.^34^ By maintaining that disabled persons should be empowered, not coerced, to determine their own lives, the Convention unequivocally challenges the paternalistic tradition embedded in the medical model.^35^ In doing so, it further provides an ethical basis for evaluating neurotechnologies: although new interventions may offer persons with disabilities fresh means of participation, the CRPD insists that such measures be undertaken with informed consent and a commitment to upholding bodily and mental integrity.

The CRPD’s transformational power does not end with its explicit demands for accessibility and anti-discrimination initiatives; it also affirms that disability is part of human diversity.^36^ This view resonates with the social model’s emphasis on celebrating, rather than merely accommodating, distinct ways of being in the world. Departing from historical efforts to ‘correct’ or eliminate impairments, the Convention’s framework highlights that structural change and adapting environments are crucial for real inclusion.^37^ Critically, it implies that emerging neurotechnologies, no matter how promising, must be formulated and applied in a way that places choice and self-determination at the center. Under this paradigm, technology does not seek to standardize disabled bodies or minds but rather to engage, respect, and empower them in all their diversity.

While the CRPD constitutes a legally binding international treaty, its domestic effect varies across legal systems. In monist jurisdictions, ratification directly incorporates the Convention into national law, whereas in dualist systems, enabling legislation is required. Nonetheless, all States Parties are obligated to ensure and promote the full realization of all human rights and fundamental freedoms for persons with disabilities within their respective legal frameworks.^38^

Viewed against the backdrop of centuries of marginalization, the CRPD’s establishment is more than a legal advance; it represents a moral and conceptual reorientation of how disability is comprehended worldwide.^39^ This broad reorientation obliges researchers, policymakers, and technological innovators to consider how their undertakings affect disability rights. Although medical interventions in the past were mostly intended to ‘cure’, current ethical imperatives demand that neurotechnological progress heed the social model’s calls: to redesign society by removing barriers, uphold the autonomy of disabled persons, and acknowledge the intrinsic worth of human difference.^40^

## IV. NEUROTECHNOLOGIES AND HUMAN RIGHTS

The evolution from the medical to the social model of disability sets the stage for a broader human-rights interrogation of neurotechnological innovation. In recent years, scholars and international institutions have explored whether existing human-rights instruments sufficiently protect the brain and mind—an inquiry that converges with, yet remains distinct from, the disability-rights approach developed under the CRPD.

Neurotechnologies occupy a pivotal place in contemporary science, providing previously inconceivable ways of accessing and modulating brain activity.^41^ Tools such as BCIs, DBS, fMRI, and EEG were initially devised to address clinical concerns, for example, epilepsy and Parkinson’s disease, or to help people with severe motor impairments.^42^ Over time, their scope has broadened to encompass consumer products, the enhancement of cognition, and even law enforcement applications.^43^

A central worry lies in the ability of these technologies to probe and interpret neural data, the bedrock of human cognition, emotion, and volition.^44^ Historically, the notion of mental privacy has referred to the moral and legal presumption that a person’s inner thoughts, emotions, and cognitive processes constitute an inviolable domain—shielded from external intrusion by virtue of the mind’s inaccessibility. In classical philosophy and early human-rights thought, this privacy of mind was implicit in the broader protection of moral autonomy and freedom of thought.^45^ Legal traditions treated the mind as the final frontier of personal integrity: an aspect of human existence presumed beyond observation, and therefore beyond regulation. Recent advances in neurotechnology, however, appear to challenge that assumption, eroding the once-natural barrier between mental life and technological reach.^46^ Contemporary researchers have demonstrated the capacity to infer subjective mental states from neural activity—identifying, for instance, whether individuals recall specific memories, perceive particular visual stimuli, or experience defined emotional valences. These developments suggest that inner cognition can now be rendered externally legible, transforming what was once considered epistemically private into a potential object of measurement and control.^47^

These developments extend beyond the realm of privacy to pose questions about mental integrity and cognitive liberty.^48^ DBS, for instance, has the power to influence emotional states or sway decision-making processes, raising fears of coercion if utilized without robust regulatory oversight.^49^ Past misapplications of medical technology—such as unethical EEG usage in disability claims^50^—illustrate the potential harm that can materialize when novel interventions exceed current regulatory capabilities or are guided by paternalistic assumptions.^51^ Meanwhile, the expansion of neurotechnologies into commercial domains highlights concerns about unequal access, where privileged demographics stand to benefit most, leaving historically marginalized communities—including those with disabilities—further behind.^52^

In response to emerging ethical risks, the concept of ‘neurorights’ has been advanced to strengthen the protection of mental and neural life.^53^ First proposed by Ienca and Andorno, this framework calls for explicit safeguards for cognitive liberty, mental privacy, mental integrity, and psychological continuity.^54^ These notions—while not new legal rights *per se*—translate existing human rights such as privacy, dignity, and autonomy into the neurotechnological domain. The debate remains unsettled: some scholars advocate codifying new rights, while others, including Bublitz,^55^ argue that existing human-rights instruments already encompass mental freedom and warn against inflating terminology. What is clear, however, is that neurotechnologies expose dimensions of human experience that demand renewed interpretive attention under international law.^56^

The following analysis situates these debates within the CRPD framework, highlighting how its principles on dignity, equality, and autonomy refine and strengthen general human-rights protections in the neurotechnological age.

## V. NEUROTECHNOLOGIES THROUGH THE LENS OF THE CRPD

Applying the CRPD to neurotechnologies thus requires interpreting its core principles—dignity, equality, autonomy, integrity, liberty, and privacy—in light of emerging scientific capacities to intervene directly in mental life.

While neurotechnologies pose significant challenges for human rights at large,^57^ they entail particularly distinct ramifications for persons with disabilities—especially individuals with intellectual or psychosocial disabilities—on account of historical abuses, the pivotal role of legal capacity, and the demands of accessibility and reasonable accommodation in decision-making processes.^58^ Building on perspectives from disability rights advocacy movements, which have identified core areas of concern rooted in longstanding struggles for autonomy, dignity, and non-discrimination, this section highlights key themes integral to the protections embodied in the CRPD. These themes mirror the disability community’s profound unease about the promises of neurotechnologies, insisting that scientific progress uphold human rights principles.

### V.A. Dignity—Beyond the Imperative of ‘Fixing’

Underlying these debates is the issue of identity, understood here as both the continuity of selfhood over time and the social recognition of difference—dimensions that neurotechnologies may unsettle by altering cognitive or affective traits central to personhood.

Contemporary convergent technologies, notably the intersection of AI and neurotechnologies, reignite the tension between ‘correcting’ disability and fostering inclusion. Devices such as cochlear implants, BCIs, and DBS can deliver unprecedented avenues for communication, mobility, and enhanced social engagement, targeting the physiological underpinnings of certain impairments and thus mitigating specific functional barriers. Yet these same devices, with their potential to modify bodies or brains directly, risk reaffirming the idea that disability is an inherent flaw requiring rectification—an outlook long entwined with eugenic discourses.^59^ The UN Special Rapporteur on the Rights of Persons with Disabilities highlights this critical concern, warning that ableism continues to shape scientific and medical advancements, reinforcing the belief that disability is a deficit to be prevented or eliminated rather than a dimension of human diversity.^60^

Underlying these debates is the issue of identity.^61^ The social model, locating disability within the interplay of individual characteristics and societal constraints, views disability as a component of human diversity, rather than a deviation demanding remedy.^62^ Neurotechnologies can complicate this understanding by offering tools to alleviate or even eliminate certain conditions.^63^ If they are adopted without due regard for the perspectives of users, such interventions may erode the autonomy of persons with disabilities and trivialize the significance of lived experience.^64^

The discourse around neurodiversity strengthens this point by emphasizing that neurological differences—such as autism or ADHD—need not be medicalized but may instead reflect naturally occurring variations in cognitive functioning.^65^ If neurotechnologies aim solely to ‘normalize’ these differences, they risk perpetuating a narrowly defined notion of social acceptability, marginalizing those whose identities fall outside normative bounds.^66^ At its core, urging that individuals conform to these expectations undermines broader efforts to establish environments that truly value and support difference.

Erving Goffman’s seminal work on stigma reveals how cultural prejudices can shape self-image, with persons with disabilities often absorbing messages that their bodies or minds are fundamentally inadequate.^67^ While AI-driven neurotechnologies may lessen some barriers, it can also reinforce stigmatizing norms—particularly if designed under the assumption that disability itself must be ‘fixed’.^68^ Such an approach risks minimizing the role of structural change, effectively placing on disabled persons the burden of ‘improvement’, rather than forcing society to dismantle exclusionary practices.^69^

The persistent legacy of eugenics accentuates the ethical imperative of circumspection.^70^ Historic examples of coerced sterilization and institutional violence underscore how readily new technologies can serve oppressive agendas.^71^ The extremes of medical paternalism found their most horrific expression in Nazi Germany’s ‘Action T4’ program, which authorized the systematic killing of more than 70,000 persons with psychosocial and intellectual disabilities under the guise of medical necessity and social hygiene. As Hassenfeld documents, the medical profession’s complicity in these atrocities demonstrates how therapeutic rhetoric can mask lethal discrimination. The memory of T4 continues to inform the disability community’s deep mistrust of technological ‘solutions’ that conflate normalization with human worth.^72^

Even modern biomedical debates—including moral questions about selective genetic screening—^73^ show how a seemingly benevolent drive to eradicate disability can inadvertently challenge the inherent worth of individuals who live with that disability.^74^

### V.B. Equality—Access, Accessibility, and Biases in Neurotechnology

Neurotechnologies hold transformative potential, yet they also raise profound concerns regarding equality, inclusion, and non-discrimination, particularly for persons with disabilities.^75^ The CRPD frames equality not merely as formal equality but as inclusive equality (Article 5), which mandates proactive measures to dismantle systemic barriers that hinder the full and equal enjoyment of rights.^76^ Within this framework, ensuring equitable access, comprehensive accessibility, and the elimination of algorithmic bias are essential pillars in advancing disability-inclusive neurotechnological innovation. Formal equality, which assumes identical treatment for all, is insufficient in the context of neurotechnologies; instead, a substantive and anticipatory approach to equality must be taken, ensuring that persons with disabilities are not merely accommodated reactively but actively considered from the outset of design, deployment, and policy implementation.^77^

Access to neurotechnology remains deeply uneven, exacerbated by economic, infrastructural, and digital divides. The financial burden of advanced neurotechnological devices, places them out of reach for many, especially in low- and middle-income contexts.^78^ These cost barriers are compounded by gaps in public funding, insurance coverage, and regulatory incentives, which often fail to prioritize assistive neurotechnologies. The digital divide further restricts access, as nearly 45 per cent of persons with disabilities face significant challenges engaging with digital technologies, limiting their ability to benefit from neurotech-driven health care, communication, and employment tools.^79^ The anticipatory obligation under the CRPD suggests that governments, developers, and policymakers must not wait for persons with disabilities to demand accommodations but must proactively design inclusive neurotechnologies. This requires integrating universal design principles from the outset, ensuring that devices and systems do not discriminate by default due to a failure to accommodate diverse needs.^80^

Accessibility is fundamental to inclusion and full participation, yet most neurotechnologies fail to incorporate universal design principles,^81^ thereby limiting their usability for persons with disabilities.^82^ The CRPD enshrines accessibility as a legal and ethical requirement (Article 9), mandating that environments, services, and technologies be designed to accommodate people with diverse impairments.^83^ However, in practice, many neurotechnological devices and systems prioritize functionality over inclusivity, resulting in barriers that disproportionately affect those who would most benefit from these innovations. Beyond the design phase, states have a legal obligation under the CRPD to foster the development and availability of accessible neurotechnologies.^84^ Article 4 explicitly mandates the promotion of research and development of assistive technologies that enhance the independence of persons with disabilities.^85^

Algorithmic bias in neurotechnologies, though often concealed within complex data processing systems, poses profound risks to disability rights and equality.^86^ AI-driven neurotechnological tools frequently inherit and reinforce existing social prejudices embedded in their training data.^87^ These biases manifest in numerous discriminatory outcomes, ranging from healthcare algorithms that fail to recognize disability-specific symptoms to recruitment platforms that penalize candidates based on atypical communication methods or motor impairments.^88^ The opacity of these AI systems, commonly known as the ‘black box’ problem, exacerbates these risks by making it difficult to scrutinize, challenge, and rectify biased decision-making processes.^89^ As a result, rather than serving as neutral instruments of progress, these technologies risk becoming mechanisms of exclusion, reinforcing systemic disparities rather than dismantling them.

Beyond exclusion, the consequences of algorithmic bias in neurotechnologies extend to the reinforcement of deeply ingrained discriminatory paradigms.^90^ AI-driven predictive analytics that combine genomic and neurological data, for instance, risk reviving eugenic ideologies by generating stigmatizing risk scores for conditions such as schizophrenia or Alzheimer’s disease—classifications that can influence critical decisions made by insurers, employers, and educational institutions.^91^ Similarly, neurotechnologies designed to forecast psychotic episodes or modify autistic behaviors often reflect ableist assumptions, prioritizing conformity to neurotypical norms over respect for neurodiversity.^92^ These applications raise urgent ethical and legal concerns about consent, privacy, and the potential coercion of individuals into involuntary behavioral modifications.^93^ As Kate Crawford emphasizes, the belief that AI can objectively categorize traits such as disability, race, or gender is fundamentally flawed, as identity is inherently dynamic and context-dependent.^94^ The misapplication of AI in neurotechnologies risks embedding reductionist classifications into decision-making processes, perpetuating a new form of digital ableism under the guise of scientific neutrality.^95^ To counteract these dangers, transparency, participatory design, and robust regulatory oversight are necessary to ensure that neurotechnologies serve as enablers of inclusion rather than instruments of discrimination.^96^

### V.C. Agency—Upholding the Right to Decide

Two distinct dimensions of agency are at stake. First, neurotechnologies may support individuals in exercising decision-making capacity—for example, through assistive communication devices. Second, individuals must retain the right to decide whether and how to use such technologies in the first place. Both dimensions require respect for autonomy and informed consent under the CRPD.

Neurotechnologies should enhance, not replace, the decision-making rights of persons with disabilities. These innovations must serve as tools for empowerment, strengthening individual autonomy rather than shifting control to guardians, medical professionals, or third parties.^97^ Historically, legal frameworks worldwide have imposed a binary model of capacity, categorizing individuals as either fully capable or legally incapacitated, often based on disability status.^98^ This has resulted in widespread substituted decision-making systems, such as guardianship, where decisions are made on behalf of individuals rather than with them.^99^ These restrictive models have disproportionately affected persons with intellectual or psychosocial disabilities, effectively stripping them of their legal agency in areas as fundamental as health care, personal finance, and civic participation.^100^

Legal capacity is a non-negotiable human right.^101^ The CRPD (Article 12) recognizes persons with disabilities as rights-holders with full legal standing, obliging states to provide access to the support persons with disabilities may require in exercizing their legal capacity. This has been interpreted as requiring supported, rather than substituted, decision-making models, among other support.^102^ This shift acknowledges that capacity is not an innate, static trait but rather a dynamic process that can be exercised with appropriate support, communication aids, and reasonable accommodations.^103^ Under this framework, all individuals retain the right to make legally binding decisions, including those related to neurotechnologies interventions affecting their bodies and minds.^104^

Neurotechnologies should never be developed or implemented in ways that bypass informed consent, impose interventions, or coerce individuals into procedures deemed ‘*in their best interest*’.^105^ The ‘*best interests*’ approach must be replaced by the ‘*will and preferences*’ model outlined in General Comment No. 1 (CRPD), which prioritizes the individual’s own choices over external judgements about their well-being.^106^ When neurotechnological tools are used without explicit user consent or in coercive circumstances, they violate fundamental human rights, including bodily integrity and freedom from exploitation (Articles 17 and 16 of the CRPD).^107^

Ensuring that legal capacity is fully respected in neurotechnologies requires a commitment to informed consent as an accessible, continuous process, providing explanations in plain language, sign language interpretation, and alternative communication formats tailored to each user’s needs.^108^ Users must be able to fully understand the risks, benefits, and alternatives before making decisions, ensuring that their consent remains free, informed, and ongoing. For many, supported decision-making is essential, requiring tailored assistance from trusted persons, peer networks, or assistive communication tools.^109^ However, this support must never amount to substituted decision-making, nor should it override the individual’s autonomy.^110^

In addition to ensuring individuals understand and navigate their choices, it is crucial that neurotechnology-assisted communication is legally recognized. Persons who rely on BCI spellers, neuro-speech devices, or AI-driven cognitive aids must have their communications respected in areas such as voting, contracts, and medical consent, ensuring that these technologies facilitate legal agency rather than restrict it.^111^ The denial of recognition for these communication methods would constitute a direct violation of the CRPD’s mandate on accessibility and legal capacity.

### V.D. Integrity—Ethical Standards in Neurotechnological Experimentation

Persons with disabilities have historically been subjected to unethical experimentation under the guise of scientific progress, a legacy that underscores the necessity of a human rights–based approach to developing and implementing neurotechnologies.^112^ Such an approach demands that neurotechnologies uphold rather than exploit the dignity, bodily integrity, and autonomy of those who may benefit from or participate in research or treatment.^113^ The imperative to avoid replicating previous injustices is firmly embedded within foundational human rights frameworks, which stress the inviolability of the person.^114^ Reflecting this emphasis, Articles 15 and 17 of the CRPD categorically prohibit non-consensual medical experimentation and actions that may constitute torture or cruel, inhuman, or degrading treatment, while also affirming each individual’s right to physical and mental integrity on an equal basis with others.^115^

A human rights-centered approach to neurotechnological research and practice requires rigorous ethical safeguards across both clinical and investigational contexts.^116^ Neurotechnological interventions possess the capacity to profoundly influence cognitive functions, behavior, and personal identity, raising critical concerns about consent, coercion, and the preservation of personal agency.^117^ Ensuring that these technologies advance individual autonomy rather than impose externally defined standards of normalcy is paramount. The disability rights movement’s demand for ‘*nothing about us without us*’ underscores that persons with disabilities must be active contributors to the development of research protocols, policy decisions, and regulatory frameworks, rather than merely passive subjects in scientific experimentation.^118^ Without meaningful participation, neurotechnological advancements risk entrenching paternalistic practices, where medical and research institutions unilaterally determine what is deemed beneficial, appropriate, or necessary for persons with disabilities, often without regard for their will and preferences.^119^

The historical misuse of psychiatric and neurological interventions illustrates the urgency of implementing heightened ethical vigilance in neurotechnologies.^120^ Contemporary neurotechnologies could present similar risks if safeguards are not stringently enforced.^121^ The use of AI-driven interventions designed to modify autistic behaviors, for example, raises concerns about social conformity being prioritized over self-determination, potentially coercing individuals to adhere to neurotypical norms rather than respecting neurological diversity.^122^ The risk of behavioral conditioning, suppression of cognitive variance, and reinforcement of ableist standards must be critically assessed to ensure that neurotechnologies serves as a tool of empowerment rather than control.^123^

The prohibition of non-consensual experimentation under the CRPD explicitly links such practices to violations of bodily and mental integrity, reinforcing the legal and ethical obligation to eliminate coercive interventions.^124^ The United Nations Special Rapporteur on Torture has affirmed that involuntary medical interventions—including forced psychiatric treatments—may constitute acts of torture when they result in severe suffering, lack medical necessity, or are carried out with discriminatory intent.^125^ These provisions impose clear obligations on states to implement strict oversight mechanisms, ensure accountability for coercive conduct, and criminalize non-consensual neurotechnological experimentation.^126^

While state-sponsored research is subject to ethical review boards, private companies are increasingly conducting human neurotechnology trials in quasi-commercial contexts. Recent examples include Neuralink’s implantation studies in the USA and Synchron’s BCI trials in Australia, both financed by major tech investors.^127^ The pace of private innovation often outstrips the capacity of national ethics systems, exposing participants—frequently persons with disabilities—to untested risks without transparent public accountability. International human-rights standards must therefore extend oversight beyond the clinical sphere to encompass corporate research and data-governance practices.

### V.E. Liberty—Safeguarding Freedom from Coercive Interventions

Forced institutionalization and coercive psychiatric interventions have long been justified under the guise of care, rehabilitation, or public safety, reinforcing a regime in which persons with disabilities, particularly those with psychosocial disabilities, are stripped of their fundamental rights.^128^ The CRPD directly challenges these practices, establishing that the existence of a disability can never justify deprivation of liberty (Article 14).^129^ This mandate disrupts the entrenched legal and medical paradigms that have historically framed psychosocial disability as a basis for detention and compelled treatment. However, this standard conflicts with other established human rights instruments, such as the International Covenant on Civil and Political Rights (ICCPR),^130^ the Council of Europe’s Oviedo Convention,^131^ and its Draft Additional Protocol,^132^ all of which permit involuntary psychiatric measures under certain conditions.^133^ This legal divergence underscores a fundamental normative conflict: while the CRPD advances an anti-discrimination model that prioritizes legal capacity and autonomy, older human rights frameworks continue to uphold public safety and medical necessity as legitimate grounds for involuntary psychiatric intervention, creating significant legal and policy tensions at both national and international levels.^134^

The persistence of legal frameworks allowing disability-based detention reflects a deep-seated paternalism that continues to permeate contemporary discourses on mental health and disability rights.^135^ The justification for psychiatric detention—whether grounded in alleged incapacity, perceived dangerousness, or the supposed necessity of medical treatment—rests on a long-discredited model that positions disabled persons as passive subjects in need of control rather than rights-bearing individuals entitled to make decisions about their own bodies and lives.^136^ As Tina Minkowitz’s critique reveals, these systems operate in tandem with broader forms of social regulation, perpetuating institutional segregation, indefinite confinement, and forced medicalization. Such practices not only deprive individuals of their liberty but also diminish their legal personhood, constructing them as objects of state and medical authority rather than as autonomous agents.^137^

A crucial dimension of the right to liberty for persons with disabilities pertains to their treatment within the criminal justice system, particularly regarding criminal responsibility.^138^ The CRPD has reshaped the debate on the criminal responsibility of persons with mental health conditions, rejecting traditional insanity defenses and unfitness to stand trial doctrines that exclude individuals from legal proceedings based on perceived incapacity.^139^ Scholars such as Minkowitz^140^ advocate for the abolition of these doctrines as inherently discriminatory, while others, like Craigie^141^ and Barsky & Stein,^142^ warn that such an approach could lead to over-criminalization and harsher penalties. In parallel, the rise of neurotechnologies in criminal investigations introduces new ethical concerns, as these tools risk reinforcing deterministic views of disability, undermining autonomy, and expanding state control over neurological data. As Gooding & O’Mahony^143^ argue, integrating neuroscience into legal proceedings without stringent human rights safeguards could entrench rather than dismantle discriminatory forensic psychiatry models.

Debates over criminal responsibility further illustrate the need for reform. Christopher Slobogin argues that disability-based legal doctrines such as the insanity defence and civil commitment should be abolished in favor of functional assessments of responsibility consistent with the CRPD’s equality model.^144^ McSherry and Simon-Butler provide a balanced overview of arguments for and against such abolition, highlighting that human-rights-based reforms must reconcile accountability with the right to equal recognition before the law.^145^ Integrating neurotechnology into forensic practice without resolving these tensions risks reproducing discrimination under a digital guise.

As Perlin and other scholars have argued, the pervasive stigma surrounding psychosocial disabilities has historically been weaponized to justify extreme restrictions on liberty, often with little regard for due process or individual rights.^146^ The introduction of neurotechnological surveillance risks entrenching these patterns by affording state and medical actors an even greater capacity to exert control over individuals deemed noncompliant or unpredictable.^147^ Under the pretext of preventing harm or ensuring adherence to treatment, such technologies could be used to impose continuous oversight, mandating compliance through remote monitoring, neural feedback mechanisms, or even direct cognitive interventions.^148^ These developments would not merely replicate past injustices but would exacerbate them, embedding coercive psychiatric control within a technologically advanced apparatus that resists scrutiny and legal accountability.^149^

The CRPD’s rejection of disability-based detention must be extended to include the emergent threats posed by neurotechnological interventions that compromise liberty under the guise of medical progress.^150^ The right to personal autonomy cannot be reconciled with regimes of perpetual monitoring and involuntary cognitive modification, particularly when these technologies operate within systems already steeped in discriminatory assumptions about disability and mental health.^151^ Without rigorous safeguards, regulatory oversight, and meaningful participatory governance that centers the perspectives of persons with disabilities, the integration of neurotechnologies into mental health and criminal justice systems risks solidifying a new era of technological oppression—one in which the deprivation of liberty is no longer confined to physical institutions but embedded in the very fabric of neurological governance.^152^

### V.F. Privacy—Protecting Personal and Neural Confidentiality

The advent of neurotechnologies and AI-driven mental health systems poses profound challenges to the right to privacy for persons with disabilities, particularly those with psychosocial or cognitive disabilities.^153^ These technologies routinely process sensitive neural, biometric, and behavioral data that can reveal core aspects of an individual’s identity, emotional state, or cognitive profile—information traditionally regarded as inviolable and private.^154^

A key dimension of this transformation is the rapid evolution and deployment of biometric technologies.^155^ It is essential to distinguish between what scholars and regulators have termed *first-generation biometrics*, typically used for *identification purposes*—such as fingerprinting, facial recognition, iris scanning, or voice authentication—and *second-generation biometrics*, which refer to the analysis of *behavioral and physiological patterns* to infer psychological states, intentions, or cognitive conditions.^156^ This second generation of biometric data, often collected passively through wearables, smartphones, or ambient sensors, includes indicators such as gait, speech cadence, eye movement, keystroke dynamics, or even heart rate variability.^157^ When paired with advanced AI systems, these markers are leveraged to construct real-time profiles of an individual’s mood, mental stability, or risk of behavioral deterioration.^158^

For persons with disabilities, the implications of these behavioral biometrics are particularly disruptive. Unlike first-generation systems, which focus on identifying who a person is, second-generation biometric systems claim to assess *what a person is thinking or feeling*—rendering the internal states of individuals legible and actionable to external systems.^159^ For people with psychosocial disabilities or neurodivergent profiles, this can mean constant exposure to systems that infer pathology from behavioral difference.^160^ Deviations from normative behavioral patterns—such as reduced eye contact, unusual voice intonation, or irregular movement—can be flagged as signs of risk or dysfunction, triggering further scrutiny or restrictive interventions.^161^ In forensic and clinical contexts, these interpretations may justify coercive measures, surveillance, or denial of services, all without the person's understanding of the criteria used or the ability to challenge the decision.

This concern is not abstract. Historical patterns of psychiatric control and custodialism have seen persons with disabilities subjected to surveillance and involuntary data extraction under the guise of treatment or safety. The transition to digital tools has not disrupted this logic but has rather recalibrated it through algorithmic systems that track, interpret, and act upon behavioral signals in real time.^162^ Digital phenotyping, for instance, converts everyday actions—such as typing cadence, phone movement, or browsing habits—into proxies for mental state assessments.^163^ These metrics are used to predict depression, anxiety, or psychotic risk without the informed and ongoing consent of the individual, and often without their knowledge. The granular intimacy of such data risks undermining personal integrity and agency, particularly when algorithmic models infer psychological states that are then used to justify surveillance, restrict opportunities, or trigger clinical or legal interventions.^164^

This becomes especially problematic in contexts already marked by unequal power relations, such as forensic psychiatry, residential institutions, or public assistance programs.^165^ In such contexts, biometric surveillance can be framed as a form of care or safety, yet often replicates the panoptic structures long critiqued in institutional psychiatry. These practices risk normalizing continuous monitoring as a condition for support, transforming what should be rights-based services into mechanisms of behavioral governance.^166^ The power asymmetries inherent in these arrangements are exacerbated by the opacity of the algorithms deployed.^167^ As machine learning models increasingly serve as the infrastructure for clinical triage, crisis intervention, or eligibility assessments, persons with disabilities face heightened vulnerability to errors, bias, and unreviewable determinations about their mental capacity, stability, or trustworthiness.^168^

At the core of these developments is the commodification of brain and behavioral data.^169^ Platforms that provide digital mental health tools or neurotechnological services^170^ often operate within opaque commercial ecosystems, where user data may be aggregated, analyzed, and monetized—frequently in ways that elude existing regulatory frameworks.^171^ This has given rise to what scholars have described as ‘vulnerability-based marketing’, in which individuals’ distress or perceived impairments are targeted to sell therapeutic apps, behavioral interventions, or data profiles to insurers, employers, or state agencies.^172^ These dynamics are particularly troubling where data are extracted from users under conditions of dependence—whether due to institutionalization, welfare conditionality, or lack of accessible alternatives—and where refusal to participate in monitoring regimes may be construed as noncompliance.^173^

The legal framework of the CRPD, particularly Article 22, establishes a firm basis for contesting these practices.^174^ It affirms the right of persons with disabilities to privacy in all matters concerning their person, including health, rehabilitation, and personal correspondence, and requires states to ensure this right on an equal basis with others.^175^ It also imposes a positive obligation to guard against arbitrary or unlawful interference, particularly in institutional settings or where individuals lack control over their environment. However, the application of Article 22 to the domain of AI and neurotechnologies remains underdeveloped, both in jurisprudence and in state practice.^176^ Despite emerging guidance from the CRPD Committee and human rights bodies, gaps persist in the regulation of biometric data, inferred mental health information, and AI-driven decision-making processes.^177^ Although instruments such as the GDPR in Europe and HIPAA in the USA regulate biometric and health data, they were not conceived to address second-generation biometrics or inferred mental-state data. These frameworks lack specific provisions on neural data derived from BCIs or AI-driven affective analytics, leaving uncertainty about consent, ownership, and cross-border data transfers. The CRPD’s Article 22 thus fills a crucial normative gap by affirming privacy not only as informational control but as a precondition for autonomy and equality.

An ongoing debate within the disability community further complicates the normative landscape. While many advocates call for stringent data protection, consent-based governance, and algorithmic transparency, others caution against framing privacy in absolute terms.^178^ They argue that data sharing, when grounded in informed agency and mutual trust, can facilitate access to essential services, community support, and collective advocacy.^179^ The challenge, then, lies in distinguishing between enabling disclosures that foster participation and coercive data regimes that instrumentalize personal information to manage, discipline, or pathologize.^180^ Bridging this divide will require a paradigm shift—from privacy as control over secrets to privacy as a condition for equality, freedom, and self-determined life in the digital age.^181^

Bridging regulatory gaps requires both substantive and procedural innovation: explicit recognition of neural data as a special category of sensitive information; mandatory human-rights impact assessments for neuro-AI systems; and participatory oversight bodies including persons with disabilities. Such measures would transform privacy from a reactive shield into an enabling condition for autonomy and equality in the digital-neural domain.

## VI. CONCLUSIONS

The convergence of neurotechnologies and disability rights presents one of the most pressing normative frontiers of the twenty-first century. As this article has argued, the challenge lies not merely in safeguarding against the material risks posed by brain-based technologies, but in ensuring that their development, deployment, and regulation are grounded in a rights-based framework capable of resisting the resurgence of paternalistic, ableist, or technocratic paradigms. The ethical terrain is complex, shaped by past abuses and future uncertainties—but also by the profound and urgent imperative to center human dignity, legal capacity, and inclusive equality.

The CRPD provides not only a normative anchor but also a radical epistemological shift. It demands that neurotechnologies be interrogated through the lived realities of persons with disabilities, whose agency, identities, and diversity must inform every stage of technological innovation. This means rejecting narratives of ‘fixing’ or ‘normalizing’ impairment, and instead embracing plural conceptions of human flourishing—rooted in the social model and enriched by the principles of accessibility, non-discrimination, and autonomy.

Moreover, the Convention’s recognition of legal capacity, bodily and mental integrity, and freedom from coercion (Articles 12, 17, and 14) imposes clear boundaries on neurotechnological practices. Devices and systems that influence cognition, behavior, or affect must operate within the paradigm of supported decision-making and informed consent. Data extraction and algorithmic surveillance—especially in forensic or clinical contexts—must not become mechanisms of digital custodialism. Instead, they must be governed by transparency, participatory design, and robust oversight that reflects the will and preferences of rights-holders.

The historical mistrust of medical institutions and scientific promises is not incidental but foundational to this conversation. It is a reminder that progress devoid of ethical reflection can re-inscribe exclusion, even under the guise of innovation. Thus, the deployment of neurotechnologies must not replicate the power asymmetries and epistemic silencing that the CRPD seeks to redress.

Finally, this article has illustrated that neurotechnologies are not inherently emancipatory or oppressive—they are tools whose implications depend on the normative frameworks that shape their use. The role of international human rights law, and particularly the CRPD, is to ensure that these tools contribute to a society in which all individuals—regardless of neurological profile—can participate fully, equally, and with dignity. This requires a collective commitment to confronting digital ableism, dismantling structural barriers, and enacting policies that respect the neurodiverse condition as an expression of human variation, not deviation.

In this sense, the CRPD is not merely a legal instrument—it is a lens through which we must evaluate the future of technological progress itself. Its ethos must guide our response to the neurotechnological age: one in which inclusion, not assimilation, marks the true measure of advancement.

## Footnotes

1 Francisco Bariffi & Gerard Quinn, *Introduction: Disability, Ethics, and Law*, in Handbook of  Disability 1 (Rioux, M.H., Buettgen, A., Zubrow, E., Viera, J. eds., 2024). https://doi.org/10.1007/978-981-16-1278-7_97-1.

2
*Global Report on Assistive Technology*. Geneva: World Health Organization (2022), https://www.unicef.org/reports/global-report-assistive-technology (accessed Mar. 22, 2025).

3
Stuart  Blume, The  Artificial  Ear: Cochlear  Implants and the  Culture of  Deafness (2010).

4 Eberhart Zrenner, *Will Retinal Implants Restore Vision?*, 295(5557) Science 1022 (2002). https://doi.org/10.1126/science.1067996.

5 Leigh R. Hochberg et al., *Reach and Grasp by People with Tetraplegia Using a Neurally Controlled Robotic Arm*, 485 Nature 372 (2012). https://doi.org/10.1038/nature11076.

6 Helen S. Mayberg et al., *Deep Brain Stimulation for Treatment-Resistant Depression*, 45(5) Neuron 651 (2005). https://doi.org/10.1016/j.neuron.2005.02.014.

7
Stuart  Murray, Disability and the  Posthuman  Bodies: Technology and  Cultural  Futures (2020). https://doi.org/10.2307/j.ctv11qdtsh.

8
Michael  Oliver, The  Politics of  Disablement: A Sociological  Approach (1990); Henri-Jacques  Stiker, A History of  Disability (2019).

9 Colin Barnes, *Understanding the Social Model of Disability: Past, Present, and Future*, in Routledge  Handbook of  Disability  Studies 12 (Watson N. Roulstone A., Thomas C. eds., 2012).

10 Francisco Bariffi, *Article 8 Awareness-Raising*, in The UN Convention on the  Rights of  Persons with  Disabilities: A Commentary 229 (Bantekas I. Stein M. A., Anastasiou D. eds., 2018). https://doi.org/10.1093/law/9780198810667.001.0001.

11
Tom  Shakespeare, Disability  Rights and  Wrongs  Revisited (2014).

12
Andrew  Scull, Madness in  Civilization: A Cultural  History of  Insanity from the  Bible to  Freud, from the  Madhouse to  Modern  Medicine (2015).

13
Anne  Borsay, Disability and  Social  Policy in  Britain since 1750: A History of  Exclusion (2005).

14
Michel  Foucault, Madness and  Civilization: A History of  Insanity in the  Age of  Reason (1988).

15 Bill Hughes & Kevin Paterson, *The Social Model of Disability and the Disappearing Body: Towards a sociology of impairment*, 12(3) Disability & Society 325 (2010). https://doi.org/10.1080/09687599727209.

16
Paul A. Lombardo, Three  Generations, No  Imbeciles: Eugenics, the  Supreme  Court, and  Buck v. Bell (2022).

17
Daniel J. Kevles, In the  Name of  Eugenics: Genetics and the  Uses of  Human  Heredity (1985).

18
Fabrice  Jotterand & Marcelo  Ienca, (ed.) Artificial  Intelligence in  Brain and  Mental  Health: Philosophical, Ethical & Policy  Issues (2023). https://doi.org/10.1007/978-3-030-74188-4.

19
Anita  Silvers, David  Wasserman & Mary B. Mahowald (eds.), Disability, Difference, and  Discrimination: Perspectives on  Justice in  Bioethics and  Public  Policy (1998); Jenny  Morris, Pride  Against  Prejudice: Transforming  Attitudes to  Disability (1991); Lennard J. Davis, The  End of  Normal: Identity in a  Biocultural  Era (2014).

20 Bernadette McSherry, *Electroconvulsive Therapy without Consent: The Influence of Human Rights Law*, 26(4) J Law  Med. 732 (2019). PMID: 31682352.

21
Fiona K. Campbell, Contours of  Ableism: The  Production of  Disability and  Abledness (2009). https://doi.org/10.1057/9780230245181.

22 Here, ‘mental autonomy’ denotes the individual’s capacity to direct one’s own thought, emotion, and decision-making processes without undue external manipulation or technological interference.

23 Gregor Wolbring, *The Politics of Ableism*, 51(2) Development 252 (2008). https://doi.org/10.1057/dev.2008.17.

24
Paddy  Ladd, Understanding  Deaf  Culture: In  Search of  Deafhood (2003).

25 Philipp Kellmeyer, *Big Brain Data: On the Responsible Use of Brain Data from Clinical and Consumer-Directed Neurotechnological Devices*, 14 Neuroethics 83 (2021). https://doi.org/10.1007/s12152-018-9371-x.

26
Joseph P. Shapiro, No  Pity: People with  Disabilities  Forging a  New  Civil  Rights  Movement (1993).

27 Paul Abberley, *The Concept of Oppression and the Development of a Social Theory of Disability*, 2(1) Disability, Handicap & Society 5 (2007). https://doi.org/10.1080/02674648766780021.

28
Colin  Barnes and  Geof  Mercer, Disability (2003).

29
Len  Barton, Disability and  Society: Emerging  Issues and  Insights (1996).

30
Gerard  Quinn and  Theresia  Degener, Human  Rights and  Disability: The  Current  Use and  Future  Potential of  United  Nations  Human  Rights  Instruments in the  Context of  Disability (2002). https://digitallibrary.un.org/record/477534?v=pdf.

31
*Convention on the Rights of Persons with Disabilities and Optional Protocol,* Dec. 13, 2006, A/RES/61/106. 2515 United  Nations, Treaty  Series 3.

32 Gerard Quinn, *The United Nations Convention on the Rights of Persons with Disabilities: Toward a New International Politics of Disability*, 15(1) Texas  Journal on  Civil  Liberties & Civil  Rights 33 (2009); Rosemary Kayess & Phillip French, *Out of Darkness into Light? Introducing the Convention on the Rights of Persons with Disabilities*, 8(1) Human  Rights  Law  Review 1 (2008). https://doi.org/10.1093/hrlr/ngm044; Agustina  Palacios, El  Modelo  Social de  Discapacidad: Orígenes, Caracterización y  Plasmación en la  Convención  Internacional sobre los  Derechos de las  Personas con  Discapacidad (2008).

33 UN Committee on the Rights of Persons with Disabilities, *General Comment No. 6 (2018) on Equality and Non-discrimination*, CRPD/C/GC/6, Apr. 26, 2018; Sandra Fredman, *Substantive Equality Revisited*, 14(3) International  Journal of  Constitutional  Law 712 (2016). https://doi.org/10.1093/icon/mow043; Francisco Bariffi, *Igualdad y no discriminación por motivo de discapacidad: hacia un modelo de igualdad inclusiva*, en Manual sobre  Justicia y  Personas con  Discapacidad 47 (Vázquez Encalada A. ed., 2021).

34 Amita Dhanda, *Legal Capacity in the Disability Rights Convention: Stranglehold of the Past or Lodestar for the Future?*, 34(2) Syracuse  Journal of  International  Law and  Commerce 429 (2006).

35 Eilionóir Flynn & Anna Arstein-Kerslake, *Legislating Personhood: Realising the Right to Support in Exercising Legal Capacity*, 10(1) International  Journal of  Law in  Context 81 (2014) 81–104. https://doi.org/10.1017/S1744552313000384.

36 Frédéric Mégret, *The Disabilities Convention: Human Rights of Persons with Disabilities or Disability Rights?*, 30(2) Human  Rights  Quarterly 494 (2008). http://www.jstor.org/stable/20072851.

37 Michael A. Stein, *Disability Human Rights*, 95(1) California  Law  Review 75 (2007).

38 Valentina Della Fina, *Article 4 [General Obligations]*, in The  United  Nations  Convention on the  Rights of  Persons with  Disabilities: A Commentary, 137–155 (V. Della Fina, R. Cera & G. Palmisano eds., Springer International Publishing, 2017). https://doi.org/10.1007/978-3-319-43790-3_5.

39 Theresia Degener, *Disability in a Human Rights Context*, 5(3) Laws 1 (2014). https://doi.org/10.3390/laws5030035.

40
Sandra  Fredman, Human  Rights  Transformed: Positive  Rights and  Positive  Duties (2008). https://doi.org/10.1093/acprof:oso/9780199272761.001.000.

41
*Novel Neurotechnologies: Intervening in the Brain*. London: Nuffield Council on Bioethics, 2013. Available at: https://cdn.nuffieldbioethics.org/wp-content/uploads/Novel-neurotechnologies-report.pdf (accessed Mar. 22, 2025).

42 Mikhail A. Lebedev & Miguel A. L. Nicolelis, *Brain–Machine Interfaces: From Basic Science to Neuroprostheses and Neurorehabilitation*, 97(2) Physiological  Reviews 767 (2017). https://doi.org/10.1152/physrev.00027.2016.

43 Allan McCay, *Neurotechnology and Human Rights: Developments Overseas and the Challenge for Australia*, 29(1) Australian  Journal of  Human  Rights 160 (2023). https://doi.org/10.1080/1323238X.2023.2221487; Marta Sosa Navarro & Salvador Dura-Bernal, *Human Rights Systems of Protection from Neurotechnologies that Alter Brain Activity*, 15(4) Drexel L. Rev. 893 (2023); Christoph Bublitz, *Neurotechnologies and Human Rights: Restating and Reaffirming the Multi-layered Protection of the Person*, 28(5) The  International  Journal of  Human  Rights 782 (2024). https://doi.org/10.1080/13642987.2024.2310830.

44 Christoph Bublitz & Reinhard Merkel, *Crimes Against Minds: On Mental Manipulations, Harms and a Human Right to Mental Self-Determination*, 8 Criminal  Law, Philosophy 51 (2014). https://doi.org/10.1007/s11572-012-9172-y.

45 Sjors Ligthart, *Mental Privacy as Part of the Human Right to Freedom of Thought?*, in The  Law and  Ethics of  Freedom of  Thought  Vol. 2: Cognitive  Liberty and  Privacy (M. Blitz, J. C. Bublitz eds., 2023).

46 Parker Crutchfield, *Mental Privacy, Cognitive Liberty, and Hog-tying*, 21 Bioethical  Inquiry 695 (2024). https://doi.org/10.1007/s11673-024-10344-0.

47 Marcello Ienca & Roberto Andorno, *Towards New Human Rights in the Age of Neuroscience and Neurotechnology*, 13(5) Life  Sciences, Society and  Policy 1 (2017). https://doi.org/10.1186/s40504-017-0050-1.

48 Christoph Bublitz, *Cognitive Liberty or the International Human Right to Freedom of Thought*, in Handbook of  Neuroethics 1309 (Clausen, J., Levy, N. eds., 2015). https://doi.org/10.1007/978-94-007-4707-4_166.

49 Philipp Kellmeyer et al., *The Effects of Closed-Loop Medical Devices on the Autonomy and Accountability of Persons and Systems*, 25(4) Camb. Q. Healthc. Ethics 623 (2016). https://doi.org/10.1017/S0963180116000359.

50 For instance, in mid-twentieth-century disability-benefit assessments in the USA, EEG readings were occasionally misused to categorize applicants as ‘uncooperative’ or ‘hysterical’, influencing benefit denial without medical basis. Jack D. Pressman, Last  Resort: Psychosurgery and the  Limits of  Medicine (1998).

51
Eric  Racine, Pragmatic  Neuroethics: Improving  Treatment and  Understanding of the  Mind-Brain (2010). https://doi.org/10.7551/mitpress/8027.001.0001.

52 Bernadette McSherry, *Neuroscience and ‘Neurointerventions’: Ethical and Legal Issues*, in Handbook of  Disability 1257 (Rioux, M.H., Buettgen, A., Zubrow, E., Viera, J. eds., 2024). https://doi.org/10.1007/978-981-19-6056-7_36.

53 Rafael Yuste et al. *Four Ethical Priorities for Neurotechnologies and AI*, 551 Nature 159 (2017). https://doi.org/10.1038/551159a.

54
*Cognitive liberty* refers to the freedom to control one’s own mental processes; *mental privacy* protects against unauthorized access to neural data or thoughts; *psychological continuity* ensures the preservation of personal identity and mental integrity over time. Marcello Ienca & Roberto Andorno, *Towards New Human Rights in the Age of Neuroscience and Neurotechnology*, 13(5) Life  Sciences, Society and  Policy 1 (2017). https://doi.org/10.1186/s40504-017-0050-1.

55 Christoph Bublitz, *Novel Neurorights: From Nonsense to Substance*, 15(7) Neuroethics 1 (2022). https://doi.org/10.1007/s12152-022-09481-3.

56
*The risks and challenges of neurotechnologies for human rights*, UNESCO, University of Milan-Bicocca—Department of Business and Law andState University of New York (SUNY) Downstate, (2023). https://doi.org/10.54678/POGS7778.

57
Jared  Genser, Stephanie  Herrmann, & Rafael  Yuste, International  Human  Rights  Protection  Gaps in the  Age of  Neurotechnology (2022). https://neurorightsfoundation.org/reports (accessed Mar. 22, 2025).

58 […Persons with disabilities are particularly exposed to medical and scientific procedures, research and experimentation. Neurotechnologies are often developed without proper human rights guidelines and monitoring, and this may expose them to a disproportionate risk of physical and/or mental harms…] *Impact, Opportunities and Challenges of Neurotechnology with Regard to the Promotion and Protection of All Human Rights*, Report of the Human Rights Council Advisory Committee, A/HRC/57/61, Aug 8, 2024.

59
Robert  Garland, The  Eye of the  Beholder: Deformity and  Disability in the  Graeco-Roman  World (1995); Colin  Barnes & Geof  Mercer, Exploring the  Divide: Illness and  Disability (1996).

60 As the report states, Such ableist ways of thinking, reinforced by the medical model, have privileged prevention and cure over all other responses to disability, leaving persons with disabilities with limited opportunities to be included and participate in society. This perspective underscores how technologies, when developed within a framework that prioritizes normalization over accommodation, can perpetuate discrimination and exclusion rather than foster true inclusion. The Special Rapporteur further warns that emerging technologies, particularly in neurotechnology and genetic interventions, risk creating a resurgence of eugenic practices, subtly devaluing the lives of persons with disabilities under the guise of medical progress. Therefore, ensuring that AI and neurotechnology uphold a rights-based, inclusive approach is imperative to prevent reinforcing historical patterns of exclusion and ableism. *Lives Not Worth Living*, Report of the Special Rapporteur on the rights of persons with disabilities, A/HRC/43/41, Dec. 17, 2019.

61
Rafael de  Asis  Roig, Sobre  Discapacidad y  Derechos (2013).

62 David-Jack Fletcher, *Transhuman Perfection: The Eradication of Disability Through Transhuman Technologies*, 7 Humana  Mente 26 (2014).

63 Rafael De Asís Roig, *Transhumanism and Disability*, in Handbook of  Disability 1 (Rioux, M.H., Viera, J., Buettgen, A., Zubrow, E. eds., 2022). https://doi.org/10.1007/978-981-16-1278-7_37-1.

64 Melinda C. Hall, *Second Thoughts on Enhancement and Disability*, in The  Oxford  Handbook of  Philosophy and  Disability 633 (Cureton A., Wasserman D. T. eds., 2019).

65 Judy Singer, *Why can’t You be Normal for Once in Yourlife? From a ‘Problem with No Name’ to a New Category of Disability*, in Disability  Discourse 59 (M. Corker, S. French eds., 1999); Thomas  Amstrong, The  Power of  Neurodiversity: Unleashing the  Advantages of  Your  Differently  Wired  Brain (2010).

66 Simon Baron-Cohen, in *The Pattern Seekers*, posits that the same genetic factors contributing to autism also underpin humanity’s unique capacity for innovation through heightened systemizing abilities. While this perspective underscores the value of neurological diversity, it has faced criticism for potentially oversimplifying the complex interplay between cognitive traits and cultural advancements. Simon  Baron-Cohen, The  Pattern  Seekers: How  Autism  Drives  Human  Invention (2020).

67
Erving  Goffman, Stigma: Notes on the  Management of  Spoiled  Identity (1963).

68
Lenard J. Davis, The  Disability  Studies  Reader (2013); Michael L. Perlin, The  Hidden  Prejudice: Mental  Disability on  Trial (2000).

69
Paul  Hunt, Stigma: The  Experience of  Disability (1966); James I. Charlton, Nothing about  Us  Without  Us: Disability  Oppression and  Empowerment (1998); Irving K. Zola, Missing  Pieces: A Chronicle of  Living with a  Disability (1998).

70
Jürgen  Habermas, The  Future of  Human  Nature (2003).

71
*Bad Blood: The Story of Eugenics*, BBC Documental Series Podcast (May 2023). https://www.bbc.co.uk/programmes/m001fd36 (accessed Mar. 22, 2025).

72 Irwin N. Hassenfeld, *Doctor–Patient Relations in Nazi Germany and the Fate of Psychiatric Patients*, 73 Psychiatric Q. 183 (2002). https://doi.org/10.1023/a:1016084620453.

73 Erik Parens & Adrienne Asch, *Disability Rights Critique of Prenatal Genetic Testing: Reflections and Recommendations*, 9(1) Ment. Retard. Dev. Disabil. Res. Rev. 40 (2003). https://doi.org/10.1002/mrdd.10056.

74 Francisco Bariffi, *Genetic Engineering and Disability: Ethical Dilemmas in the Verge of Artificial Evolution*, in Handbook of  Disability 1 (Rioux, M.H., Buettgen, A., Zubrow, E., Viera, J. eds., 2024). https://doi.org/10.1007/978-981-19-6056-7_38; Javier Romañach Cabrero, *Diversity Ethics and the Impact of Genetic Technologies*, in Genetic  Discrimination: Transatlantic  Perspectives on the  Case for a  European  Level  Legal  Response (G. Quinn, A. de Paor, P. Blanck eds., 2014). https://doi.org/10.4324/9780203674299.

75 Gerard Goggin, *Disability, Internet, and Digital Inequality: The Research Agenda*, in Disability, Internet, and digital inequality: the research agenda 255 (Hargittai E. ed., 2021). https://doi.org/10.4337/9781788116572; Tetyana (Tanya) Krupiy & Martin Scheinin, *Disability Discrimination in the Digital Realm: How the ICRPD Applies to Artificial Intelligence Decision-Making Processes and Helps in Determining the State of International Human Rights Law*, 23(3) Human  Rights  Law  Review 1 (2023). https://doi.org/10.1093/hrlr/ngad019; Virginia  Eubanks, Automating  Inequality: How  High-Tech  Tools  Profile, Police, and  Punish the  Poor (2019).

76 Committee on the Rights of Persons with Disabilities, *General Comment No. 6 (2018) on Equality and Non-discrimination*, CRPD/C/GC/6, Apr. 26, 2018.

77 […Without meaningful engagement and participation of persons with disabilities in their design, development and implementation, neurotechnologies risk contributing to stigmatization and objectification, reinforcing structural-based discrimination…] *Impact, Opportunities and Challenges of Neurotechnology with Regard to the Promotion and Protection of All Human Rights*, Report of the Human Rights Council Advisory Committee, A/HRC/57/61, Aug. 8, 2024; *General Comment No. 7 (2018) on the Participation of Persons with Disabilities, including Children with Disabilities, through Their Representative Organizations, in the Implementation and Monitoring of the Convention*, Committee on the Rights of Persons with Disabilities, CRPD/C/GC/7, Nov. 9, 2018; Gregor Wolbring, *Neuro-Abilities and a Good Life*, 14(1) J. Neurol. Res. 16 (2024). https://doi.org/10.14740/jnr770.

78
*Digital Progress and Trends Report 2023*, The World Bank (2024).

79
Jan van  Dijk, The  Digital  Divide (2020); Cynthia K. Sanders & Edward Scanlon, *The Digital Divide Is a Human Rights Issue: Advancing Social Inclusion Through Social Work Advocacy*, 6 J. Hum. Rights  Soc. Work 130 (2021). https://doi.org/10.1007/s41134-020-00147-9.

80 Office of the United Nations High Commissioner for Human Rights, *Mapping Report: Human Rights and New and Emerging Digital Technologies*, A/HRC/56/45, Aug. 20, 2024, ¶ 43.

81 Universal design, as defined in art. 2 of the CRPD, entails the creation of products, environments, and services usable by all people, to the greatest extent possible, without the need for adaptation. Complementary frameworks such as the WHO’s Global Cooperation on Assistive Technology (GATE) and UNESCO’s 2021 Recommendation on the Ethics of Artificial Intelligence further emphasize accessibility, interoperability, and participatory design as essential components of inclusive innovation.

82 L. Elisabeth Armstrong, *Accounting for Diversity, Inclusion, and Accessibility in Early-Stage Neurotechnology Research*, 3(4) GEN Biotechnology 215 (2024). https://doi.org/10.1089/genbio.2024.0012.

83 Committee on the Rights of Persons with Disabilities, *General Comment No. 2 Article 9: Accessibility*, CRPD/C/GC/2, May 22, 2014.

84
Michael  Ashley  Stein & Jonathan  Lazar, (eds.) Accessible  Technology and the  Developing  World (2022).

85
Elizabeth R. Petrick, Making  Computers  Accessible: Disability  Rights and  Digital  Technology (2015).

86
Meredith  Whittaker et al., Disability, Bias, and AI (2019). https://ainowinstitute.org/publication/disabilitybiasai-2019 (accessed Mar. 22, 2025).

87
Cathy O’Neil, Weapons of  Math  Destruction: How  Big  Data  Increases  Inequality and  Threatens  Democracy (2017).

88 Francisco Bariffi, *Artificial Intelligence, Human Rights and Disability*, 26(2) Revista  Pensar de  Ciências  Jurídicas 1 (2021). https://doi.org/10.5020/2317-2150.2021.12704.

89
*Artificial Intelligence and the Rights of Persons with Disabilities*, Report of the Special Rapporteur on the rights of persons with disabilities, A/HRC/49/52, Dec. 28, 2021.

90 Arleen Salles & Michele Farisco, *Neuroethics and AI Ethics: A Proposal for Collaboration*, 25 BMC Neuroscience 45 (2024). https://doi.org/10.1186/s12868-024-00888-7.

91 Uchenna E. Okpete & Haewon Byeon, *Challenges and Prospects in Bridging Precision Medicine and Artificial Intelligence in Genomic Psychiatric Treatment*, 14(8) World J. Psychiatry 1148 (2024). https://doi.org/10.5498/wjp.v14.i8.1148.

92 Sara Goering & Eran Klein. *Neurotechnologies and Justice by, with, and for Disabled People*, in The Oxford Handbook of Philosophy and Disability 616 (Cureton A., Wasserman D. T. eds., 2019). https://doi.org/10.1093/oxfordhb/9780190622879.013.33.

93 Nicholas Tilmes, *Disability, Fairness, and Algorithmic Bias in AI Recruitment*, 24 Ethics  Inf  Technol 21 (2022).

94
Kate  Crawford, Atlas of AI: Power, Politics, and the  Planetary  Costs of  Artificial  Intelligence (2022).

95
*Lives Not Worth Living*, Report of the Special Rapporteur on the rights of persons with disabilities, A/HRC/43/41, Dec. 17, 2019.

96 Gerard Goggin, *Disability and Digital Inequalities: Rethinking Digital Divides with Disability Theory*, in Theorizing  Digital  Divides (M. Ragnedda, G. W. Muschert eds., 2017). https://doi.org/10.4324/9781315455334.

97 Piers Gooding, *Navigating the ‘Flashing Amber Lights’ of the Right to Legal Capacity in the United Nations Convention on the Rights of Persons with Disabilities: Responding to Major Concerns*, 15(1) Human  Rights  Law  Review 45 (2015). https://doi.org/10.1093/hrlr/ngu045.

98 Francisco Bariffi, *Capacidad jurídica y capacidad de obrar de las personas con discapacidad a la luz de la Convención de la ONU*, in Hacia un derecho de la discapacidad: estudios en homenaje al profesor  Rafael de  Lorenzo (L. Cayo Pérez Bueno dir. 2009).

99
Francisco  Bariffi, El  Régimen  Jurídico  Internacional de la  Capacidad  Jurídica de las  Personas con  Discapacidad (2014).

100 Anna Arstein-Kerslake & Jennifer Black, *Right to Legal Capacity in Therapeutic Jurisprudence: Insights from Critical Disability Theory and the Convention on the Rights of Persons with Disabilities*, 68 International  Journal of  Law and  Psychiatry 101535 (2020). https://doi.org/10.1016/j.ijlp.2019.101535; Robert Dinerstein, *Implementing Legal Capacity Under Article 12 of the UN Convention on the Rights of Persons with Disabilities: The Difficult Road from Guardianship to Supported Decision-Making*, 19(2) Human  Rights  Brief 8 (2012). https://ssrn.com/abstract=2040938.

101
*General Comment No. 1. Article 12: Equal Recognition before the Law*, Committee on the Rights of Persons with Disabilities, CRPD/C/GC/1, May 19, 2014.

102 Bernadette McSherry, *Legal Capacity Under the Convention on the Rights of Persons with Disabilities*, 20 Journal of  Law and  Medicine 22 (2012). https://ssrn.com/abstract=2490972.

103 Antonio Martinez-Pujalte, *Legal Capacity and Supported Decision-Making: Lessons from Some Recent Legal Reforms*, 8(1) Laws 4 (2019). https://doi.org/10.3390/laws8010004.

104 Eilionoir Flynn & Anna Arstein-Kerslake, *Legislating Personhood: Realising the Right to Support in Exercising Legal Capacity*, 10(1) International  Journal of  Law in  Context 81 (2014). https://doi.org/10.1017/S1744552313000384.

105 Bernadette McSherry, *Decision-Making, Legal Capacity and Neuroscience: Implications for Mental Health Laws*, 4(2) Laws 125 (2015). https://doi.org/10.3390/laws4020125.

106 […Where, after significant efforts have been made, it is not practicable to determine the will and preferences of an individual, the ‘best interpretation of will and preferences’ must replace the ‘best interests’ determinations. This respects the rights, will and preferences of the individual, in accordance with art. 12, ¶ 4. The ‘best interests’ principle is not a safeguard which complies with art. 12 in relation to adults. The ‘will and preferences’ paradigm must replace the ‘best interests’ paradigm to ensure that persons with disabilities enjoy the right to legal capacity on an equal basis with others…] *General Comment No. 1. Article 12: Equal Recognition before the Law*, Committee on the Rights of Persons with Disabilities, CRPD/C/GC/1, May 19, 2014, ¶ 21.

107 Jane Buchanan & Marine Uldry, *Legal Capacity: Personal Choice and Control*, in 8 European  Human  Rights  Report, EDF (2024).

108 Liz Brosnan & Eilionóir Flynn, *Freedom to Negotiate: A Proposal Extricating ‘Capacity’ from ‘Consent’*, 13(1) International  Journal of  Law in  Context 58 (2017). https://doi.org/10.1017/S1744552316000471.

109
Michael  Bach & Lana  Kerzner, A New Paradigm for Protecting Autonomi and the Right to Legal Capacity. Advancing  Substantive  Equality for  Persons with  Disabilities through  Law, Policy and  Practice (2010). https://hdl.handle.net/11250/3032345 (accessed Mar. 22, 2025).

110 Richard Lombard-Vance et al., *Applying the ‘Human Rights Model of Disability’ to Informed Consent: Experiences and Reflections from the SHAPES Project*, 3(1) Disabilities 28 (2023). https://doi.org/10.3390/disabilities3010003.

111 Sjors Ligthart et al., *Minding Rights: Mapping Ethical and Legal Foundations of ‘Neurorights’*, 32(4) Cambridge  Quarterly of  Healthcare  Ethics 461 (2023). https://doi.org/10.1017/S0963180123000245.

112
Thomas S. Szasz, The  Myth of  Mental  Illness: Foundations of a  Theory of  Personal  Conduct (1972); Erving  Goffman, Asylums: Essays on the  Social  Situation of  Mental  Patients and  Other  Inmates (1961).

113 Piers Gooding, *Supported Decision-Making: A Rights-Based Disability Concept and Its Implications for Mental Health Law*, 20(3) Psychiatry, Psychology and  Law 431 (2013). https://doi.org/10.1080/13218719.2012.711683.

114
Manfred  Nowak, U.N. Covenant on  Civil and  Political  Rights: CCPR Commentary (2005); UN Human Rights Committee, *CCPR General Comment No. 20: Article 7 (Prohibition of Torture, or Other Cruel, Inhuman or Degrading Treatment or Punishment)*, Mar. 10, 1992. https://www.refworld.org/legal/general/hrc/1992/en/11086.

115 Francesco Seatzu, *Article 17 Protecting the Integrity of the Person*, in The UN Convention on the  Rights of  Persons with  Disabilities: A Commentary 494 (Bantekas I. Stein M. A., Anastasiou D., eds., 2018). https://doi.org/10.1093/law/9780198810667.003.0018; Mary Keys, *Article 17 Protecting the Integrity of the Person*, in The  United  Nations  Convention on the  Rights of  Persons with  Disabilities: A Commentary 327 (Della Fina, V., Cera, R., Palmisano, G. eds., 2017). https://doi.org/10.1007/978-3-319-43790-3_21.

116 Christoph Bublitz, *Neurotechnologies and Human Rights: Restating and Reaffirming the Multi-layered Protection of the Person*, 28(5) The  International  Journal of  Human  Rights 782 (2024). https://doi.org/10.1080/13642987.2024.2310830; Andrés Constantin, *Human Subject Research: International and Regional Human Rights Standards*, 20(2) Health  Hum  Rights 137 (2018). PMID: 30568408.

117 Sara Goering & Rafael Yuste, *On the Necessity of Ethical Guidelines for Novel Neurotechnologies*, 167(4) Cell 882 (2016). https://doi.org/10.1016/j.cell.2016.10.029.

118
James  Charlton, Nothing  About  Us  Without  Us: Disability  Oppression and  Empowerment (1998). https://doi.org/10.1525/california/9780520207950.001.0001.

119 Mark Priestley, Lisa Waddington & Carlotta Bessozi, *Towards an Agenda for Disability Research in Europe: Learning from Disabled People’s Organisations*, 25(2) Disability & Society 731 (2010). https://doi.org/10.1080/09687599.2010.505749; Anna Nilsson & Linus Broström, *Participation in Research and the CRPD*, 25 International  Journal of  Mental  Health and  Capacity  Law 1 (2019). https://doi.org/10.19164/ijmhcl.v2019i25.937.

120 Stephen Rainey & Yasemin J. Erden, *Correcting the Brain? The Convergence of Neuroscience, Neurotechnology, Psychiatry, and Artificial Intelligence*, 26 Sci  Eng  Ethics 2439 (2020). https://doi.org/10.1007/s11948-020-00240-2.

121 Sara Goering et al., *Staying in the Loop: Relational Agency and Identity in Next-Generation DBS for Psychiatry*, 8(2) AJOB Neuroscience 59 (2017). https://doi.org/10.1080/21507740.2017.1320320; Vera Tesink et al., *Right to Mental Integrity and Neurotechnologies: Implications of the Extended Mind Thesis*, 50(10) Journal of  Medical  Ethics 656 (2024). https://doi.org/10.1136/jme-2023-109645.

122 Andrew Fenton & Tim Krahn, *Neurodiversity, and Equality Beyond the ‘Normal’,* 2(2) Journal of  Ethics in  Mental  Health 1 (2007).

123 Marcelo Ienca & Karolina Ignatiadis, *Artificial Intelligence in Clinical Neuroscience: Methodological and Ethical Challenges*, 11(2) AJOB Neuroscience 77 (2020). https://doi.org/10.1080/21507740.2020.1740352.

124 […As has been stated by the Committee in several concluding observations, forced treatment by psychiatric and other health and medical professionals is a violation of the right to equal recognition before the law and an infringement of the rights to personal integrity (art. 17); freedom from torture (art. 15); and freedom from violence, exploitation and abuse (art. 16). (…) Forced treatment is a particular problem for persons with psychosocial, intellectual, and other cognitive disabilities. States parties must abolish policies and legislative provisions that allow or perpetrate forced treatment, as it is an ongoing violation found in mental health laws across the globe, despite empirical evidence indicating its lack of effectiveness and the views of people using mental health systems who have experienced deep pain and trauma as a result of forced treatment…] *General Comment No. 1. Article 12: Equal Recognition before the Law*, Committee on the Rights of Persons with Disabilities, CRPD/C/GC/1, May 19, 2014.

125 In 2008, Manfred Nowak emphasized that forced psychiatric treatments could constitute torture or ill-treatment, especially when lacking medical necessity or conducted with discriminatory intent. He noted that such practices might inflict severe mental and physical suffering. *Interim Report of the Special Rapporteur on Torture and Other Cruel, Inhuman or Degrading Treatment or Punishment*, A/63/175, July 28, 2008.

126 In 2013, Juan E. Méndez highlighted that non-consensual medical treatments, including forced psychiatric interventions, can amount to torture or cruel, inhuman, or degrading treatment. He stressed that these practices violate the principles of respect for autonomy and physical and mental integrity. Report of the Special Rapporteur on torture and other cruel, inhuman or degrading treatment or punishment, Juan E. Méndez, A/HRC/22/53, Feb. 1, 2013.

127 Allan McCay, *Neurotechnology and Human Rights: Developments Overseas and the Challenge for Australia*, 29 Austl. J. Hum. Rts. 160 (2023).

128
Thomas  Szasz, Coercion as  Cure: A Critical  History of  Psychiatry (2007); Thomas  Szasz, Liberation by  Oppression: A Comparative  Study of  Slavery and  Psychiatry (2002); Michael Evans et al. *Coercion in Psychiatry and Mental Health Care: Ethical, Legal, and Human Rights Considerations*, 6(3) World  Social  Psychiatry 112 (2024). https://doi.org/10.4103/wsp.wsp_21_24; Dirk Richter, *Coercion in Psychiatry: The Human Rights Challenge*, in Coercion and  Violence in  Mental  Health  Settings 173 (Hallett, N., Whittington, R., Richter, D., Eneje, E. eds., 2024). https://doi.org/10.1007/978-3-031-61224-4_8.

129
*Guidelines on the Right to Liberty and Security of Persons with Disabilities*, Report of the Committee on the Rights of Persons with Disabilities, A/72/55, 2017. These Guidelines challenge long-standing mental health laws, criminal responsibility doctrines, and coercive psychiatric practices, demanding full legal capacity, voluntary medical treatment, and access to justice on an equal basis with others. The guidelines also highlight emerging threats, such as the use of neurotechnologies in criminal justice and mental health monitoring, reinforcing that any interference with mental autonomy must be subject to strict human rights protections.

130 The ICCPR, through art. 9, guarantees the right to liberty and security, prohibiting arbitrary detention but historically allowing involuntary psychiatric commitment under certain conditions. Before the adoption of the CRPD, interpretations of the ICCPR permitted mental health-based detention if an individual was deemed a danger to themselves or others or if commitment was considered medically necessary under parens patriae principles. The 1991 UN Principles for the Protection of Persons with Mental Illness (MI Principles A/RES/46/119) reinforced this approach, emphasizing procedural safeguards but still allowing involuntary admission when risk or medical deterioration was present. This medicalized perspective stands in direct conflict with the CRPD, which rejects all disability-based detention as discriminatory. In 2014, the Human Rights Committee issued a new interpretation of art. 9 of the ICCPR, notably omitting any reference to disability rights or the CRPD standard. *General Comment No. 35 Article 9 (Liberty and Security of Person)*, Human Rights Committee, CCPR/C/GC/35, Dec. 16, 2014.

131 The Oviedo Convention (Convention on Human Rights and Biomedicine—COE—ETS No 164-) establishes free and informed consent as the standard for medical interventions (art. 5) but makes an exception for persons with serious mental disorders under art. 7, allowing involuntary treatment if necessary to prevent serious harm. This provision reflects a medicalized approach to mental health, prioritizing clinical necessity over personal autonomy, and stands in clear tension with the CRPD, which categorically prohibits disability-based deprivation of liberty.

132 The objective of this new Protocol is to develop, for the first time in a legally binding instrument, the provisions of art. 7 of the Convention on Human Rights and Biomedicine, as well as those of art. 5 § 1 (e) of the European Convention on Human Rights. The Protocol aims at setting out the fundamental guarantees with regard to this very exceptional possibility of interference in the rights to freedom and autonomy of persons. *Draft Additional Protocol to the Convention on Human Rights and Biomedicine Concerning the Protection of Human Rights and Dignity of Persons with Regard to Involuntary Placement and Involuntary Treatment within Mental Healthcare Services*, CM(2022)14-add1 25/01/2022. While the draft protocol introduces procedural safeguards, it continues to allow forced psychiatric interventions under certain conditions. This approach has faced strong opposition from disability rights organizations and CRPD advocates, who argue that it perpetuates coercive practices and contradicts the CRPD’s absolute rejection of involuntary psychiatric commitment. Overview of issues created by the Draft Additional Protocol to the Council of Europe’s Oviedo Convention in light of the CRPD, European Disability Forum, Nov. 02, 2021, https://www.edf-feph.org/content/uploads/2021/11/Overview-of-issues-with-Oviedo-Draft-Protocol-in-light-of-CRPD.pdf.

133 János Fiala-Butora, *The Influence of the Convention on the Rights of Persons with Disabilities on the European Court of Human Rights in the Area of Mental Health Law: Divergence and Enexplored Potential*, 94 International  Journal of  Law and  Psychiatry 101965 (2024). https://doi.org/10.1016/j.ijlp.2024.101965.

134 Elsewhere, I have argued that in cases of normative conflict between the CRPD and earlier human rights instruments, the CRPD standard should take precedence under international treaty law, applying the principles of lex specialis (the more specific law prevails over the general) and lex posteriori (the later law supersedes the earlier). *Id.* at. 95, chap. 6.

135 Anna Arstein-Kerslake, *Challenging the Foundations of Mental Health Law: Using Articles 12 and 14 of the CRPD as a Framework to Deconstruct and Reimagine Mental Health Law*, in The  Future of  Mental  Health, Disability and  Criminal  Law (Wilson K., Maker Y., Gooding P., Walvisch J. eds., 2023).

136 Amita Dhanda, *From Duality to Indivisibility: Mental Health Care and Human Rights*, 32(3) South  African  Journal on  Human  Rights 438 (2017). https://doi.org/10.1080/02587203.2016.1263416; Francisco Bariffi & Matthew Smith, *Same Old Game but with Some New Players—Assessing Argentina’s National Mental Health Law in Light of the Rights to Liberty and Legal Capacity under the UN Convention on the Rights of the Persons with Disabilities*, 31(3) Nordic  Journal of  Human  Rights, 325 (2013); Charles O’Mahony, *Disability, Mental Health, and International Human Rights Law: A Global Health Perspective*, in The  Routledge  International  Handbook of  Disability and  Global  Health (N. Lieketseng, M. Velarde, S. Singh, L. Swartz, K. Soldatić eds., 2024). https://doi.org/10.4324/9781003228059.

137 Tina Minkowitz, *The United Nations Convention on the Rights of Persons with Disabilities and the Right to Be Free from Nonconsensual Psychiatric Interventions*, 34(2) Syracuse  Journal of  International  Law and  Commerce 405 (2007). https://ssrn.com/abstract=1481512.

138 […In the area of criminal law, recognition of the legal capacity of persons with disabilities requires abolishing a defence based on the negation of criminal responsibility because of the existence of a mental or intellectual disability.41 Instead disability-neutral doctrines on the subjective element of the crime should be applied, which take into consideration the situation of the individual defendant. Procedural accommodations both during the pretrial and trial phase of the proceedings might be required in accordance with article 13 of the Convention, and implementing norms must be adopted…] *Thematic Study by the Office of the United Nations High Commissioner for Human Rights on Enhancing Awareness and Understanding of the Convention on the Rights of Persons with Disabilities*, A/HRC/10/48, Jan. 26, 2009, ¶ 47.

139 The CRPD Committee’s Guidelines on the Right to Liberty and Security of Persons with Disabilities explicitly rejects findings of unfitness to stand trial and the insanity defense, arguing that such legal doctrines deprive individuals of due process and equal legal protections. It maintains that all persons with disabilities accused of crimes must be afforded the same procedural rights as other defendants, with reasonable accommodations to enable their effective participation in legal proceedings. The Guidelines also call for eliminating security measures, such as forensic psychiatric detention and compulsory treatment orders, emphasizing that these practices amount to arbitrary deprivation of liberty. *Guidelines on the Right to Liberty and Security of Persons with Disabilities*, Report of the Committee on the Rights of Persons with Disabilities, A/72/55, 2017.

140 Tina Minkowitz, *Rethinking Criminal Responsibility from a Critical Disability Perspective: The Abolition of Insanity/Incapacity Acquittals and Unfitness to Plead, and Beyond*, 23(3) Griffith  Law  Review 434 (2014). https://doi.org/10.1080/10383441.2014.1013177.

141 Jillian Craigie, *Against a Singular Understanding of Legal Capacity: Criminal Responsibility and the Convention on the Rights of Persons with Disabilities*, 40 International  Journal of  Law and  Psychiatry 6 (2015). http://dx.doi.org/10.1016/j.ijlp.2015.04.002.

142 Benjamin A. Barsky & Michael A. Stein, *The United Nations Convention on the Rights of Persons with Disabilities, Neuroscience, and Criminal Legal Capacity*, 10(1) Journal of  Law and the  Biosciences 1 (2023). https://doi.org/10.1093/jlb/lsad010.

143 Piers Gooding & Charles O'Mahony, *Laws on Unfitness to Stand Trial and the UN Convention on the Rights of Persons with Disabilities: Comparing Reform in England, Wales, Northern Ireland and Australia*, 44 International  Journal of  Law, Crime and  Justice 122 (2016). https://doi.org/10.1016/j.ijlcj.2015.07.002.

144 Christopher Slobogin*, Eliminating Mental Disability as a Legal Criterion in Deprivation of Liberty Cases: The Impact of the Convention on the Rights of Persons With Disabilities*, 40 Int’l J. L. & Psychiatry 36 (2015). https://doi.org/10.1016/j.ijlp.2015.04.011.

145 Bernadette McSherry & Andrew Simon-Butler, *Human Rights Law and the Defence of Mental Impairment*, 29 J. L. & Med. 62 (2022).

146 Michael L. Perlin, *International Human Rights and Institutional Forensic Psychiatry: The Core Issues*, in The  Use of  Coercive  Measures in  Forensic  Psychiatric  Care 9 (Völlm, B., Nedopil, N. eds., 2016). https://doi.org/10.1007/978-3-319-26748-7_2; Michael L. Perlin, *Understanding the Intersection between International Human Rights and Mental Disability Law: The Role of Dignity*, in The  Routledge Handbook of International Crime and Justice Studies (B. Arrigo & H. Bersot eds., 2013).

147 Piers Gooding & Yvette Maker, *The Digital Turn in Mental Health and Disability Law*, in The  Future of  Mental  Health, Disability and  Criminal  Law (K. Wilson, Y. Maker, P. Gooding, J. Walvisch eds., 2024). https://doi.org/10.4324/9781003350644-17.

148 Piers Gooding, *Mapping the Rise of Digital Mental Health Technologies: Emerging Issues for Law and Society*, 67 International  Journal of  Law and  Psychiatry 101498 (2019). https://doi.org/10.1016/j.ijlp.2019.101498.

149 Sarah Carr, *‘AI Gone Mental’: Engagement and Ethics in Data-Driven Technology for Mental Health*, 29(2) Journal of  Mental  Health 125 (2020). https://doi.org/10.1080/09638237.2020.1714011.

150 Kevin Chien-Chang Wu, *Addressing Disability in Global Mental Health and Neuroethics: Challenges and Hopes*, 15 Global  Mental  Health and  Neuroethics 237 (2020). https://doi.org/10.1016/B978-0-12-815063-4.00015-0.

151 Laura Davidson, *A Key, Not a Straitjacket: The Case for Interim Mental Health Legislation Pending Complete Prohibition of Psychiatric Coercion in Accordance with the Convention on the Rights of Persons with Disabilities*, 22(1) Health  Hum  Rights (2020). PMID: 32669798.

152 Sara Goering, Timothy Brown & Eran Klein, *Neurotechnology Ethics and Relational Agency*, 16(4) Philosophy  Compass (2021). https://doi.org/10.1111/phc3.12734.

153 Piers Gooding, *Power, Personhood, and Data-Driven Technologies in the Lives of Disabled People: The Rise of Profiling Technologies in Mental Health Settings*, in Handbook of  Disability 1297 (Rioux, M.H., Buettgen, A., Zubrow, E., Viera, J. eds., 2024). https://doi.org/10.1007/978-981-19-6056-7_87.

154
Carissa  Véliz, Privacy  Is  Power (2020); Patrick Magee, Marcello Ienca & Nita Farahany, *Beyond Neural Data: Cognitive Biometrics and Mental Privacy*, 112(8) Neuron 3017 (2024). https://doi.org/10.1016/j.neuron.2024.09.004; […The operation of AI systems can facilitate and deepen privacy intrusions and other interference with rights in a variety of ways. These include entirely new applications as well as features of AI systems that expand, intensify or incentivize interference with the right to privacy, most notably through increased collection and use of personal data…] *The Right to Privacy in the Digital Age*, Report of the United Nations High Commissioner for Human Rights, A/HRC/48/31, Sep. 13, 2021.

155
Anil K. Jain, Patrick  Flynn, & Arun A. Ross (eds.), Handbook of  Biometrics (2008). https://doi.org/10.1007/978-0-387-71041-9.

156 Margit Sutrop & Katrin Laas-Mikko, *From Identity Verification to Behavior Prediction: Ethical Implications of Second Generation Biometrics*, 29(1) Review of  Policy  Research 21 (2012). https://doi.org/10.1111/j.1541-1338.2011.00536.x.

157
Emilio  Mordini & Dimitros  Tzovaras (eds.), Second  Generation  Biometrics: The  Ethical, Legal and  Social  Context (2012). https://doi.org/10.1007/978-94-007-3892-8.

158 Nicole Martinez-Martin et al., *Data Mining for Health: Staking Out the Ethical Territory of Digital Phenotyping*, 1 npj  Digital  Med 68 (2018). https://doi.org/10.1038/s41746-018-0075-8.

159
Nita A. Farahany, The  Battle for  Your  Brain: Defending the  Right to  Think  Freely in the  Age of  Neurotechnology (2023).

160 Sam Perochon et al., *Early Detection of Autism Using Digital Behavioral Phenotyping*, 29 Nat  Med 2489 (2023). https://doi.org/10.1038/s41591-023-02574-3.

161
Lydia X. Z. Brown et al., Ableism  and  Disability  Discrimination  in  New  Surveillance  Technologies  How New Surveillance Technologies in Education, Policing, Health Care, and the Workplace Disproportionately Harm Disabled People (2022). https://cdt.org/wp-content/uploads/2022/05/2022-05-23-CDT-Ableism-and-Disability-Discrimination-in-New-Surveillance-Technologies-report-final-redu.pdf (accessed Mar. 22, 2025).

162
Carissa  Véliz, The  Ethics of  Privacy and  Surveillance (2023); Antoine Oudin et al., *Digital Phenotyping: Data-Driven Psychiatry to Redefine Mental Health*, 25 J Med  Internet  Res (2023). https://doi.org/10.2196/44502.

163 Nicole Martinez-Martin, Henry T. Greely & Mildred K. Cho, *Ethical Development of Digital Phenotyping Tools for Mental Health Applications: Delphi Study*, 9(7) JMIR Mhealth  Uhealth (2021). https://doi.org/10.2196/27343.

164 Giovanni Stanghellini & Federico Leoni, *Digital Phenotyping: Ethical Issues, Opportunities, and Threats*, 11 Front. Psychiatry (2020). https://doi.org/10.3389/fpsyt.2020.00473.

165 Alex Z.R. Chia & Melvyn W.B. Zhan, *Digital Phenotyping in Psychiatry: A Scoping Review*, 30(6) Technology and  Health  Care (2022). https://doi.org/10.3233/THC-213648.

166 Sonia Cea Quintana, *Liberal Democracy, Behavioural Governance, and Communication: Risks and Opportunities*, in Data-Driven  Governance  Through AI, Digital  Marketing, and the  Privacy  Interplay (J. Ramón Saura ed., 2023). https://doi.org/10.4018/979-8-3693-6945-6.

167 Mason Marks, *Algorithmic Disability Discrimination*, in Disability, Health, Law, and  Bioethics (I. Cohen, C. Shachar, A. Silvers, M. A. Stein eds., 2020). https://doi.org/10.1017/9781108622851.

168 Frank Pasquale, *The Algorithmic Self*, 17(1) Hedgehog  Review (2015).

169 Rafael Yuste, *Advocating for Neurodata Privacy and Neurotechnology Regulation,* 18 Nat  Protoc 2869 (2023). https://doi.org/10.1038/s41596-023-00873-0.

170 Bernadette McSherry, *Computational Modelling, Social Media and Health-Related Datasets: Consent and Privacy Issues*, 25(4) J. Law  Med. 894 (2018). PMID: 29978673.

171 Francis X. Shen et al., *Returning Individual Research Results from Digital Phenotyping in Psychiatry*, 24(2) The  American  Journal of  Bioethics 69 (2023). https://doi.org/10.1080/15265161.2023.2180109.

172
*Consumer Vulnerability in the Digital Age*: OECD Digital Economy Papers, No. 355, OECD Publishing (2023). https://www.oecd.org/content/dam/oecd/en/publications/reports/2023/06/consumer-vulnerability-in-the-digital-age_85b498eb/4d013cc5-en.pdf.

173 John Torous et al., *The Growing Field of Digital Psychiatry: Current Evidence and the Future of Apps, Social Media, Chatbots, and Virtual Reality*, 20(3) World  Pshychatry 318 (2021). https://doi.org/10.1002/wps.20883.

174 Mark C. Weber, *Protection for Privacy under the United Nations Convention on the Rights of Persons with Disabilities*, 6(3) Laws 10 (2017). https://doi.org/10.3390/laws6030010.

175 Valentina Della Fina, *Article 22 Respect for Privacy*, in The  United  Nations  Convention on the  Rights of  Persons with  Disabilities: A Commentary 401 (2017). https://doi.org/10.1007/978-3-319-43790-3_26.

176 IDA’s Compilation of CRPD Committee’s Concluding Observations: Article 22 CRPD (Respect for privacy), September 2023. https://www.internationaldisabilityalliance.org/documents/article22crpd3-pdf; Article 22: Illustrative indicators on respect for privacy, OHCHR, UN. https://www.ohchr.org/sites/default/files/documents/issues/disability/sdg-crpd-resource/indicators/article-22-indicators-edited-dm-template.pdf.

177 […despite the mental health benefits that neurotechnologies will bring, there is a fear that neurodata will not only allow us to know what people are thinking (which is not possible for now), but also to manipulate the human brain…] *Foundations and Principles for the Regulation of Neurotechnologies and the Processing of Neurodata from the Perspective of the Right to Privacy*, Report of the Special Rapporteur on the right to privacy, Ana Brian Nougrères, A/HRC/58/58, Jan. 16, 2025.

178 Daniel Mont, *Ethical and Legal Issues in the Collection, Use, and Dissemination of Data on Disability*, in Advances in  Disability  Research  Ethics 33 (Good, A., Elliott, I. and Mallon, S. eds., 2024). https://doi.org/10.1108/S2398-601820240000011003.

179 Jonathan Lazar, Brian Wentz & Marco Winckler, *Information Privacy and Security as a Human Right for People with Disabilities*, in Accessible  Technology and the  Developing  World (M. A. Stein, J. Lazar eds., 2022). https://doi.org/10.9783/9780812294095-014.

180
Silvia  Yee & Mary  Lou  Breslin, This  Data, Not  That  Data: Big  Data, Privacy, and the  Impact on  People with  Disabilities (2023). https://healthlaw.org/wp-content/uploads/2023/03/This-Data-Not-That-Data_Disability-Rights-Education-and-Defense-Fund_FINAL.pdf (accessed Mar. 22, 2025).

181 Abhimanyu Vasudeva, Nishat A. Sheikh & Samantak Sahu, *Digitization of Disability Certification Using Technology to Curtail Infringement of Dignity*, 10 J Family  Med  Prim  Care 3948 (2021). https://doi.org/10.4103/jfmpc.jfmpc_961_21.

